# Specific human gene expression in response to infection is an effective marker for diagnosis of latent and active tuberculosis

**DOI:** 10.1038/s41598-024-77164-5

**Published:** 2024-11-06

**Authors:** Ritah Nakiboneka, Natasha Walbaum, Emmanuel Musisi, Michael Nevels, Tonney Nyirenda, Marriott Nliwasa, Chisomo L. Msefula, Derek Sloan, Wilber Sabiiti

**Affiliations:** 1 Division of Infection and Global Health, School of Medicine, University of St Andrews, St Andrews, KY16 9TF UK; 2 Department of Pathology, Kamuzu University of Health Sciences, Blantyre, Malawi; 3 Helse Nord Tuberculosis Initiative (HNTI), Pathology Department, Kamuzu University of Health Sciences, Blantyre, Malawi; 4 Africa Centre for Public Health and Herbal Medicines (ACEPHEM), Kamuzu University of Health Sciences, Blantyre, Malawi; 5 Adroit Biomedical and Bio-entrepreneurship Research Services (ABBRS), Kampala, Uganda; 6 Biomedical Sciences Research Complex (BSRC), School of Biology, University of St Andrews, St Andrews, UK

**Keywords:** Host gene expression, Diagnosis, Active tuberculosis, Latent tuberculosis, Reverse transcriptase-quantitative PCR, Transcriptomics, Molecular medicine

## Abstract

RNA sequencing and microarray analysis revealed transcriptional markers expressed in whole blood can differentiate active pulmonary TB (ATB) from other respiratory diseases (ORDs), and latent TB infection (LTBI) from healthy controls (HC). Here we describe a streamlined reverse transcriptase quantitative polymerase chain reaction (RT-qPCR) assay that could be applied at near point-of-care for diagnosing and distinguishing ATB from ORDs and LTBI from HC. A literature review was undertaken to identify the most plausible host-gene markers (HGM) of TB infection. Primers, and dual labelled hydrolysis probes were designed and analytically evaluated for accuracy in an in-vitro model of infection using a lung fibroblast cell-line. Best performing genes were multiplexed into panels of 2–4 targets and taken forward for clinical evaluation. Mycobacteria Growth Indicator Tube and QuantiFERON-TB Gold Plus were used as reference tests for ATB and LTBI respectively. A total of 16 HGM were selected and incorporated into five multiplex RT-qPCR panels. PCR assay efficiency of all evaluated targets was ≥ 90% with a median analytical sensitivity of 292 copies/µl [IQR: 215.0-358.3 copies/µl], and a median limit of quantification of 61.7 copies/µl [IQR: 29.4-176.3 copies/µl]. Clinically, ATB was characterised by higher gene expression than ORDs, while LTBI was associated with lower gene expression than HC, Kruskal-Wallis p < 0.0001. Crucially, BATF2, CD64, GBP5, C1QB, GBP6, DUSP3, and GAS6 exhibited high differentiative ability for ATB from ORDs, LTBI or HC while KLF2, PTPRC, NEMF, ASUN, and ZNF296 independently discriminated LTBI from HC. Our results show that different HGM maybe required for ATB and LTBI differentiation from ORDs or HC respectively and demonstrate the feasibility of host gene-based RT-qPCR to diagnose ATB and LTBI at near point-of-care.

**Supplementary Information:**

The online version contains supplementary material available at 10.1038/s41598-024-77164-5.

## Introduction

Early accurate detection of tuberculosis (TB) permits prompt treatment, breaks the chain of transmission, improves treatment outcome, and reduces related morbidity and mortality^1^. However, currently approved methods of TB diagnosis have posed several challenges to diagnosis and management of TB leading the World Health Organization (WHO) to call for non-sputum triage tests for TB diagnosis^2^. Transcriptional markers differentially expressed (DE) in active pulmonary TB (ATB) disease have been identified and are being evaluated as possible non-sputum alternatives for TB diagnosis^3^. Transcriptomic discovery studies used micro-bead arrays^4–8^and RNA sequencing^9^ to identify DE genes in ATB compared to healthy controls (HC), latently infected TB (LTBI) or participants with other respiratory diseases (ORDs). These discovery studies reported hundreds^8,10^ to thousands^5,6^ of host gene markers (HGM) and required sophisticated programming and trained experts to analyse the data. Complex analysis, however, makes clinical application of the findings difficult and conversion of the technology underpinning this approach to more straightforward near point-of-care (POC) tests with an easily interpretable read-out was required.

**Figure 1:**
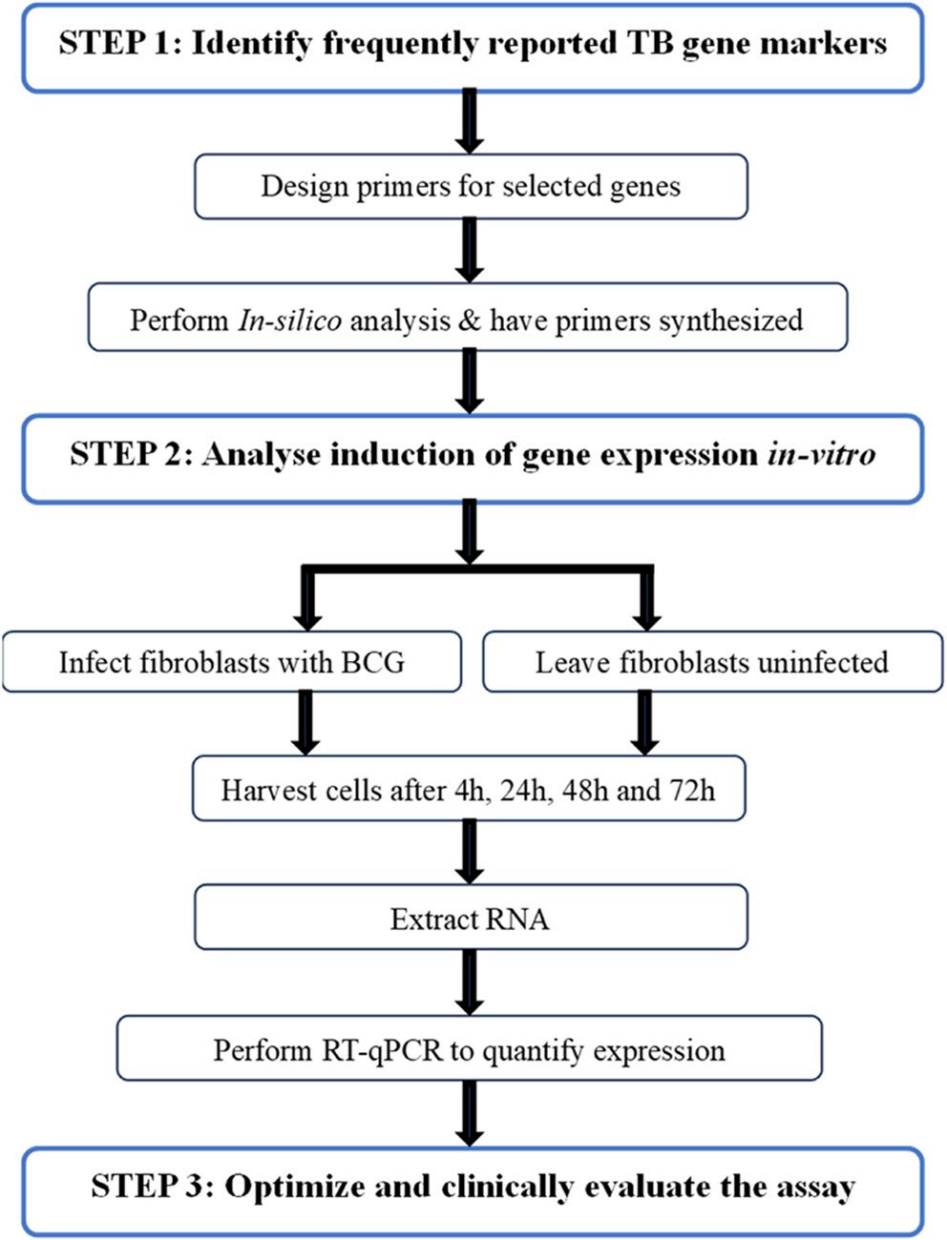
Schematic flow diagram illustrating steps taken to design the RT-qPCR assay. BCG- bacillus Calmette–Guérin, RNA- ribonucleic acid and RT-qPCR- reverse transcriptase quantitative polymerase chain reaction assay.

Reverse transcriptase quantitative polymerase chain reaction (RT-qPCR) assays allow for HGM quantification in whole blood after an initial RNA extraction step. This technology received great pertinency during the Coronavirus Infectious Disease 2019 (COVID-19) pandemic and was considered the gold standard for diagnosis of the infection^11^. Consequently, there is now increased worldwide availability of real-time qPCR machines^12^in clinical facilities, alongside skilled technical personnel to operate them. Several groups have used the RT-qPCR technology for ATB diagnosis through transcriptional marker quantification, although assay efficiency and quantitative read-outs are rarely reported^12–15^. Also, microarray quantifiable DE gene levels may not directly translate to measurable targets in RT-qPCR, as Darboe et al. reported some microarray discovered DE gene targets showing inconsistence in microfluidic RT-qPCR^16^. Here, we describe the steps taken to develop, optimise and evaluate an RT-qPCR assay to detect and quantify selected host transcriptional markers of TB infection (illustrated in Fig. 1) and report the assay quantitative read-out in well-characterised participant groups.

## Materials and methods

### Identification of host-gene markers for evaluation and design of qPCR primers, and probes

Published scientific literature was searched for plausible TB gene signatures using the Google Scholar search engine in March of 2021. The search term used was “tuberculosis transcriptional signatures”. Twenty signatures were selected as shown in Supplementary Table 1. Genes in the 20 signatures were listed, analysed, and scored for frequency of citation in the literature. Based on frequency of citation in the different selected signatures, as well as predicted network associations and biological function analysis performed on the STRING database^17^ in the same period, genes were selected for inclusion into the customised RT-qPCR assay. Three reference genes namely *ACTB*, *GAPDH* and *B2M*reported highly expressed in fibroblasts by Vandesompele et al.^18^ were also added to the panel and evaluated for invariance.

Primer design for the selected gene markers was performed using the Primer Quest program on the Integrated DNA Technologies (IDT) platform^19^. In-silico analysis of the selected primer pairs for hairpin formation, primer dimer formation and heterodimer formation were performed using the IDT Oligo Analyzer tool^20^. Primer sequencies for *GBP5*, *DUSP3* and *KLF2*were identical to those published by Francisco et al.^12^, and suitable probes matching the primer sequencies were designed for these genes using the same IDT platform.

### Assessing primer performance in gene expression quantification

Human embryonic lung fibroblast (MRC-5 cells, ATCC-CCL-171) were infected with Bacillus Calmette–Guérin (BCG) a surrogate of *Mycobacterium tuberculosis* (MTB) to induce gene expression in an in-vitro experiment. Cells used in the in-vitro experiment were at passages 24, 26, and 27 propagated in Dulbecco’s Modified Eagle Medium supplemented with 10% foetal bovine serum (FBS). Each well of a six-well culture plate was seeded with 1.2 × 10^6^ MRC-5 cells. These were incubated at 37^o^C and in 5% CO_2_ for 3 days to reach confluence before infection. Four wells of each culture plate were inoculated with 100 µl of BCG at optical density of 0.4 which is equivalent to 10^5^ estimated colony forming units (eCFU) per ml and no inoculum was added to two control wells. Plates were then incubated under 5% CO_2_ in a humidified incubator with sampling (cell harvesting) done at 4, 24, 48 and 72 h. Cell activity at each sampling point was arrested using a phenol-containing lysis buffer.

RNA extraction from MRC-5 cells was performed using the phenol-chloroform procedure. Briefly, the lysed mixture was transferred to 300 µl of chloroform, vortexed and centrifuged. The upper aqueous phase, containing the nucleic acids was harvested into new tubes. Ice-cold 100% absolute ethanol was added to precipitate the nucleic acids. The pellet was further washed with 70% ethanol, dried in a heat hot block at 50^o^C for 30 min and resuspended in RNase-free water. Genomic DNA was removed from the RNA extract by treating the extract with RNase-free DNase set from Qiagen for 40 min at 30^o^C. The reaction was stopped by placing the tubes on ice.

Host gene RT-qPCR was performed on a Rotor-Gene 5plex platform (Qiagen, UK) using HGM specific primers and QuantiTect SYBR Green (Qiagen) reagent. Briefly, the SYBR green cycle included an initial step of reverse transcription (RT) at 50^o^C for 20 min followed by a polymerase activation step for 15 min at 95^o^C. This was followed by 40 cycles each having a step of denaturation at 94^o^C for 15 s, annealing at 60^o^C for 30 s and final extension at 72^o^C for 20 s. For each gene reaction, a RT negative tube was included to confirm complete removal of genomic DNA. Data were analysed using the Rotor-Gene Q pure detection software Version 2.3.5 (Build 1).

### Amplification efficiency of the designed primers

To determine primer amplification efficiency of the target genes in relation to the reference ACTB, two RNA extracts from 72 h-BCG-infected MRC-5 cells were used. Sample RNA concentration was determined using a Nanodrop machine. The samples were diluted in a 10-fold seven times. Each target dilution was normalized with a similar dilution of the reference gene. The ΔCq values were then plotted on a line graph on the basis that an absolute slope value close to zero would indicate similar amplification efficiencies of the target and reference genes and mean that the primer sets were an efficient pair^21^.

### Quantification of bacterial load in harvested infected media and cells

Lysis of both cell-free bacilli in the media and cell-associated bacilli was performed using the phenol-chloroform procedure. To each sample, 100 µl of a known concentration extraction control was added. The infected media was vortexed, spun down, and the pellet re-suspended in FASTRNA Pro blue solution (lysis) buffer. The mixture was transferred to lysing matrix made of 0.5 μm zirconium beads and homogenized at 6,000 rpm for 40 s using, Percellys 24 (Bertin Germany) homogeniser. After a step of centrifugation at 12,000 g for 5 min, the supernatant was harvested into 300 µl of chloroform followed by RNA purification steps as described in the RNA extraction step above. Quantification of bacilli was performed on a Rotor-Gene 5plex platform (Qiagen, UK) using primers, dual-labelled hydrolysis probes (TaqMan) targeting MTB complex 16 S rRNA, and the extraction control and QuantiTect Multiplex RT-PCR kit (Qiagen) as described by Sabiiti et al.^22^.

### Primer probe concentration determination and assay multiplexing

In-silico analysis for the selected probe sequencies was performed to confirm that the primers and probe combinations would not anneal to each other. Only four colour detection channels were possible, thus designed probes were based on these. These included fluorescein amidites (FAM) acquired on the green channel, ATTO700 acquired on the crimson channel, ROX acquired on the orange channel and Hexachloro-fluorescein (HEX) which was acquired on the yellow channel.

The multiplex assay included HGM specific primers and TaqMan dual labelled hydrolysis probes specific for the target genes and was also performed on a Rotor-Gene 5plex platform. Concentrations of the primers and probes used were determined by (1) changing concentrations for each primer and its probe in a single target reaction to determine optimum concentration, (2) combining the primers and probes in a single, duplex, and finally multiplex reaction.

The RT-qPCR cycle published by Sabiiti et al.^23^ was adopted. It included, an initial reverse transcription step at 50^o^C for 30 min, DNA polymerase activation step at 95^o^C for 15 min followed by 40 amplification cycles each having a step of denaturation at 94^o^C for 45 s and annealing at 60^o^C for 60 s.

### Target standard reconstitution, standard curve creation and PCR efficiency determination

Procured from Eurofins genomics (Germany) in a lyophilised form, primer-probe target region standards were diluted to a starting concentration of 100 pmol/µl. Using the supplied molecular weight (MW) for each target, conversion to ng/µl was performed. Final concentration conversion to copies/µl for all the targets was performed using Avogadro’s constant (6.02E + 23)^24^, base pair length of the nucleic acid, conversion factor to ng (1.00E + 09) and molecular weight of a DNA base pair (660). The formula below was used for the calculation:

$$\mathrm{Copies}/\mathrm\mu\;\mathrm l=\frac{\mathrm{Amount}\;(\mathrm{ng})\ast\mathrm{Avogadro}'s\;\mathrm{constant}}{\mathrm{Length}\;(\mathrm{bp})\ast1.00\mathrm E+09\;\ast660}$$

Target standards were diluted to a working stock of 1:10 and mixed within their allocated panels described above. Fourteen further 10-fold dilutions were made to create the standard curve. Amplification efficiency curves of the standards were drawn to determine the assay dynamic range and PCR efficiency. Neat samples to the 1:10,000 dilution for all targets did not yield good sigmoid amplification since the targets were too concentrated. Good sigmoid shape amplification curves were observed from the 1:100,000 dilution. To determine the limit of detection (LOD) and limit of quantification (LOQ), amplification efficiency assays of the target standard were performed in a 10-fold dilution 7 times, starting from a concentration of 2.8E + 08 copies/µl (1 × 10^−5^ dilution) to a 2.8E-01 copies/µl (1 × 10^−14^ dilution).

### Clinical evaluation of the assay in human participants

Between 4th Jan 2022 and 30th September 2022, participants with Xpert MTB/RIF- and Mycobacteria Growth Indicator Tube (MGIT) culture- confirmed pulmonary ATB were prospectively enrolled into the study from three healthcare facilities (HCFs) in Blantyre Malawi. Participants with TB-like symptoms but testing negative for both Xpert MTB/RIF and MGIT culture were also enrolled into the study from the same HCFs, and these were denoted ORDs participants. ORDs were tested for LTBI using Gold plus QuantiFERON (QFT-Plus) test. Household contacts of confirmed ATB cases were enrolled and those who tested positive for TB Gold plus QuantiFERON (QFT-Plus) test were classified as LTBI. HC were recruited from the same Blantyre community based on HIV negative status, no known TB exposure, and a negative QFT-Plus test. Individuals were excluded from the study if they did not consent to join, and if they were less than 18 years old.

### Sputum liquid culture and xpert MTB/RIF tests

MTB detection in clinical samples was performed using MGIT culture and Xpert MTB/RIF Ultra test. To eliminate non-MTB bacteria before MGIT culture, sputum samples were decontaminated with 3% Sodium hydroxide + N-acetyl-L-cysteine (NaoH + Nalc) in a 1:1 sample-reagent ratio for 15 min. The action of NaoH + Nalc was neutralized by addition of sterile phosphate buffered saline. The mixture was centrifuged at 3000 g for 20 min to concentrate the bacteria and get rid of excess supernatant. Each MGIT tube (Becton Dickinson (BD) supplemented with BACTEC MGIT 960 OADC Growth Supplement (BD) and MGIT lyophilised mixture of Polymyxin B, Amphotericin B, Nalidixic Acid, Trimethoprim and Azlocilin (PANTA) Antibiotic Mixture (BD) was inoculated with 0.5mls of the concentrated decontaminated sample. Tubes were then incubated in the MGIT culture instrument at 37^o^C until they flagged positive in a 42-day period. Samples that did not flag positive within 42 days, were considered TB negative. Presence of MTB species in positive cultures was confirmed by acid fast bacilli (AFB) Ziehl-Neelsen (ZN) staining and detection of antigen MPT64, a protein that is only produced by *Mycobacterium tuberculosis* complex. For the Xpert MTB/RIF Ultra test, samples were mixed with sample reagent in a 1:2 volume ratio to liquefy the sputum and left to stand for 15 min at room temperature. The mixture was then added to the Xpert MTB/RIF Ultra cartridge and run on the GeneXpert instrument.

### RNA extraction, quantification and QFT-Plus assay

Procedures for RNA extraction, quantification, and QFT-Plus have previously been described^25^. Briefly, peripheral whole blood was collected from all study participants into Paxgene tubes and QFT-Plus tubes to perform these procedures. RNA extraction was performed using the PAXgene blood RNA kit (PreAnalytiX cat.no 762174) as per the manufacturer’s recommendation. Extracted RNA yield was measured using Qubit RNA High Sensitivity reagent (Invitrogen Cat. No Q32855) on the Qubit machine version 3.0 (Life Technologies)^26^ whilst the QFT-Plus test was performed following the Qiagen 2018 kit insert^27^.

### Statistical analysis

Data analysis was performed using Microsoft Excel and R statistical programming (version 4.2.1, Boston)^28^. For the cell infection assay, data normalisation was performed by subtracting the average Cycle quantification (Cq) values of the reference gene ACTB from the average Cq value of the target gene^29^. Whilst for clinical evaluation, Cq results were converted to concentration using target specific standard curves and recorded as copies/µl. Differences between groups were performed using Mann-Whitney U test, Kruskal-Wallis test and Dunn’s Test with Bonferroni corrected p-values for multiple comparisons. A p-value of < 0.05 was considered significant with *=<0.05, **=<0.01, ***=<0.001, and ****=<0.0001. Assay LOD and LOQ were determined as described by Bustin et al.^30^ and Kralik et al.^31^ respectively. Quantitative read-outs of the RT-qPCR assay data are reported as median with interquartile ranges [IQR]. Data visualisation was performed using GraphPad Prism (version 10.0.0 Boston, Massachusetts USA) and RStudio 2023.06.1 Build 524 (Posit Software, PBC)^28^.

### Ethical approval

This work was approved by the University of St Andrews Teaching and Research Ethics Committee (UTREC) under approval code MD15741 and the College of Medicine Research Ethics committee (COMREC) Malawi under Protocol number P.06/21/3342. All participants in the clinical evaluation step agreed to join the study and provided written informed consent. All study procedures and experiments were conducted in accordance with the relevant guidelines and regulations.

## Results

### Selection of a plausible panel of genes to convert into a diagnostic test for TB

The 20 selected signatures (details in Supplementary Table 1) comprised of 549 genes (excluding repeats). Of these, 490/549 appeared in only one signature, 41/549 appeared in 2 signatures and 11/549 appeared in 3 signatures. *GBP5* was the most frequently reported gene appearing in 8/20 signatures with a 40% occurrence followed by *GBP6* at a 35% occurrence (7/20 signatures). *C1QB*, *FCGR1B*, *SEPT4*, *FCGR1A*, and *GAS6* followed with a 25% or 20% frequency of occurrence in published literature. Therefore, all these HGM were included in the panel. Table 1 provides details of reported gene markers with the highest frequency of occurrence.

**Table 1:** Gene occurrence in 20 selected TB reported signatures.

No.	Gene	No. of times mentioned	% Occurrence
1	*GBP5*	8	40
2	*GBP6*	7	35
3	*C1QB*	5	25
4	*FCGR1B / CD64B*	5	25
5	*SEPT4*	5	25
6	*FCGR1A / CD64A*	4	20
7	*GAS6*	4	20
8	*ANKRD22*	3	15
9	*BATF2*	3	15
10	*C1QC*	3	15
11	*DHRS9*	3	15
12	*DUSP3*	3	15
13	*FER1L3 (MYOF)*	3	15
14	*GBP1*	3	15
15	*IFITM3*	3	15
16	*SERPING1*	3	15
17	*SMARCD3*	3	15
18	*STAT1*	3	15

**Figure 2:**
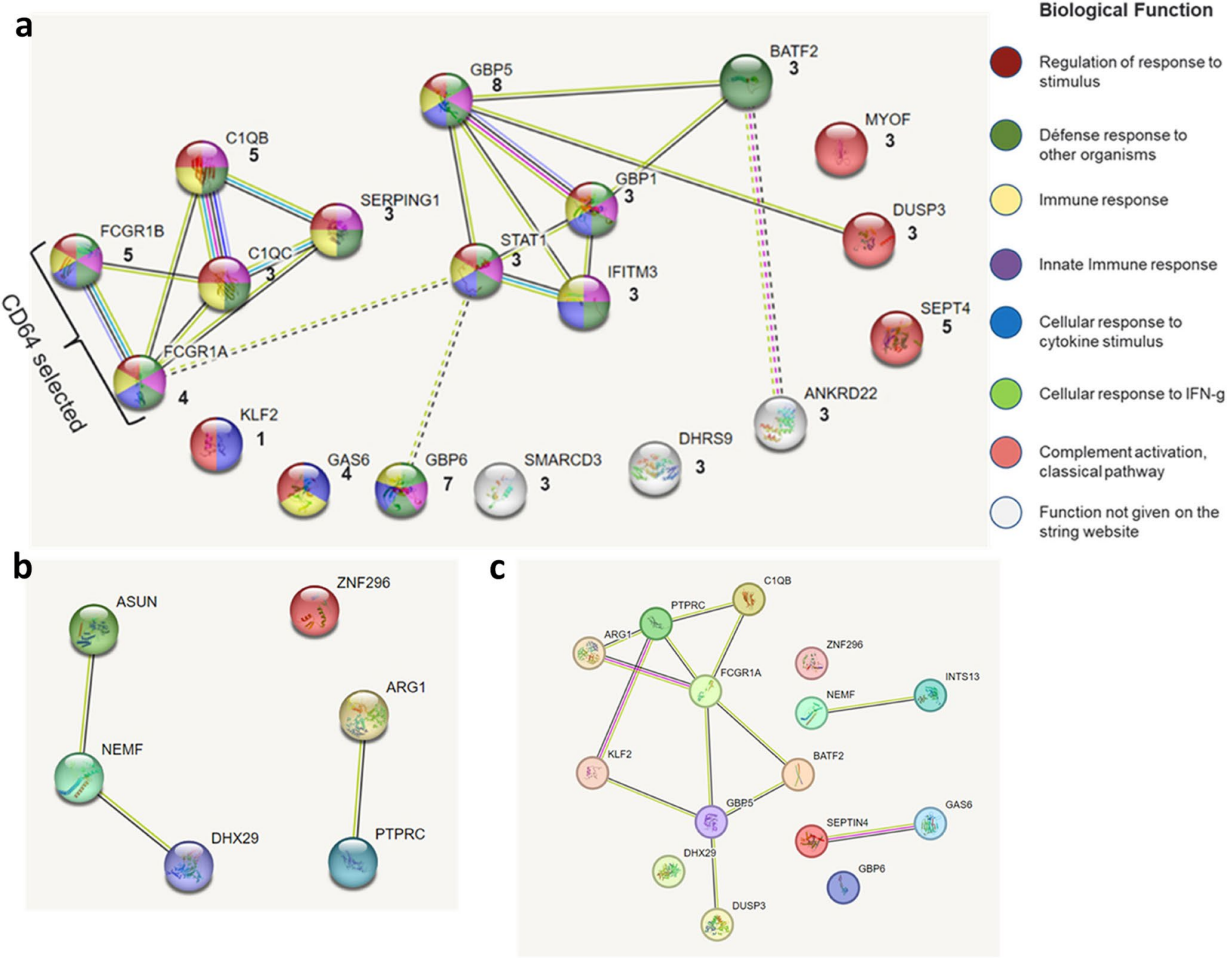
Frequently reported TB gene markers and functional relationships between them. (**A**) Selected gene biomarker protein interaction network created on the STRING website. Genes were grouped according to their associated pathways and biological functions. The colour within the circles represents the biological function and the key summarises the functions. The evidence mode was used to summarize the network predicted associations. Lines indicate the available evidence used in predicting the functional associations, the colour of the lines denotes the type of interaction evidence. The number of interaction lines illustrate more evidence of modules available for that interaction. Seven types of interaction evidence were used. Interaction analysis on the STRING website was performed in March of 2021 and may change with new evidence submitted to the database. Numbers underneath the gene symbol indicate the number of times gene was mentioned in plausible signatures. (**B**) Network interaction of the additional markers for LTBI. (**C**) Final interaction network of all the selected gene markers.

Next, the selected genes and those that occurred in at least 3 signatures (11 genes) were submitted to the STRING database^17^ to ascertain protein-protein interaction clustering and biological function. Selection among the eleven genes reported in 3 different signatures was based on a protein having unique biological function different from the above already selected frequently occurring (No. 1–7). Proteins clustering with the already selected most frequently occurring markers, and having similar biological function as those prior selected markers were excluded. Figure 2a shows the modelling performed on the STRING website and the clustering. Consequently, *BATF2* and *DUSP3* were additionally selected and added to the panel since they had unique biological function compared to *GBP5* with only two predicted network interactions. Furthermore, since the Sweeney3 signature^32^ now had 2 of its genes selected, *KLF2* was added to this panel to make the 3 gene Sweeney3 panel complete. Since *GBP1*,* IFITM3* and *STAT1* were in the same cluster of biological functions as *GBP5*, they were excluded. Similarly, *C1QC* and *SERPING1* were clustering with the complement marker *C1QB* and were therefore excluded. The biological functions of *ANKRD22*, *DHRS9* and *SMARCD3* were not given on the STRING website and were thus also excluded. Lastly, *MYOF* was excluded because it had similar function as *DUSP3*. Primer design and in-silico analysis proceeded for the selected panel. *CD64* primers were designed to span both the *FCGR1B* and *FCGR1A* regions. Primer pairs for reference genes *ACTB*, *GAPDH* and *B2M* were also designed. Further research was performed to identify plausible markers highly discriminatory of LTBI from HC and a panel of *ASUN*, *NEMF*, *DHX29* and *PTPRC* published by Lee et al.^15^ was added. HGM *ZNF296* and *ARG1* reported downregulated in ATB disease compared to LTBI and ORDs, respectively^33^, were also added to the panel. Figure 2b shows the additional marker panel. A protein network showing the selected 15 markers for evaluation is shown in Fig. 2c. Annotation of the selected gene biological functions as indicated in literature are shown in Supplementary Table 2.

### Designed primers and probes were optimal for accurate amplification

The designed primer length was between 18 and 25mer. Guanine-cytosine (GC) content was 40 to 66.7%. All hairpins formed by the primers had a melting temperature (Tm) below the Tm of the primers, all the primers had a Gibbs free energy (∆G) for formation of heterodimers of greater than − 10 and none formed strong heterodimer bonds with each other. Supplementary Table 3 summarises key characteristics for each primer. Similarly, designed probe properties are included in Supplementary Table 4. These did not form heterodimers with the primers, were 0 to 6 base pairs longer than the primers and had slightly higher Tm than the primers.

### Successful infection of MRC-5 cells and characterisation of primer performance in HGM quantification

**Figure 3:**
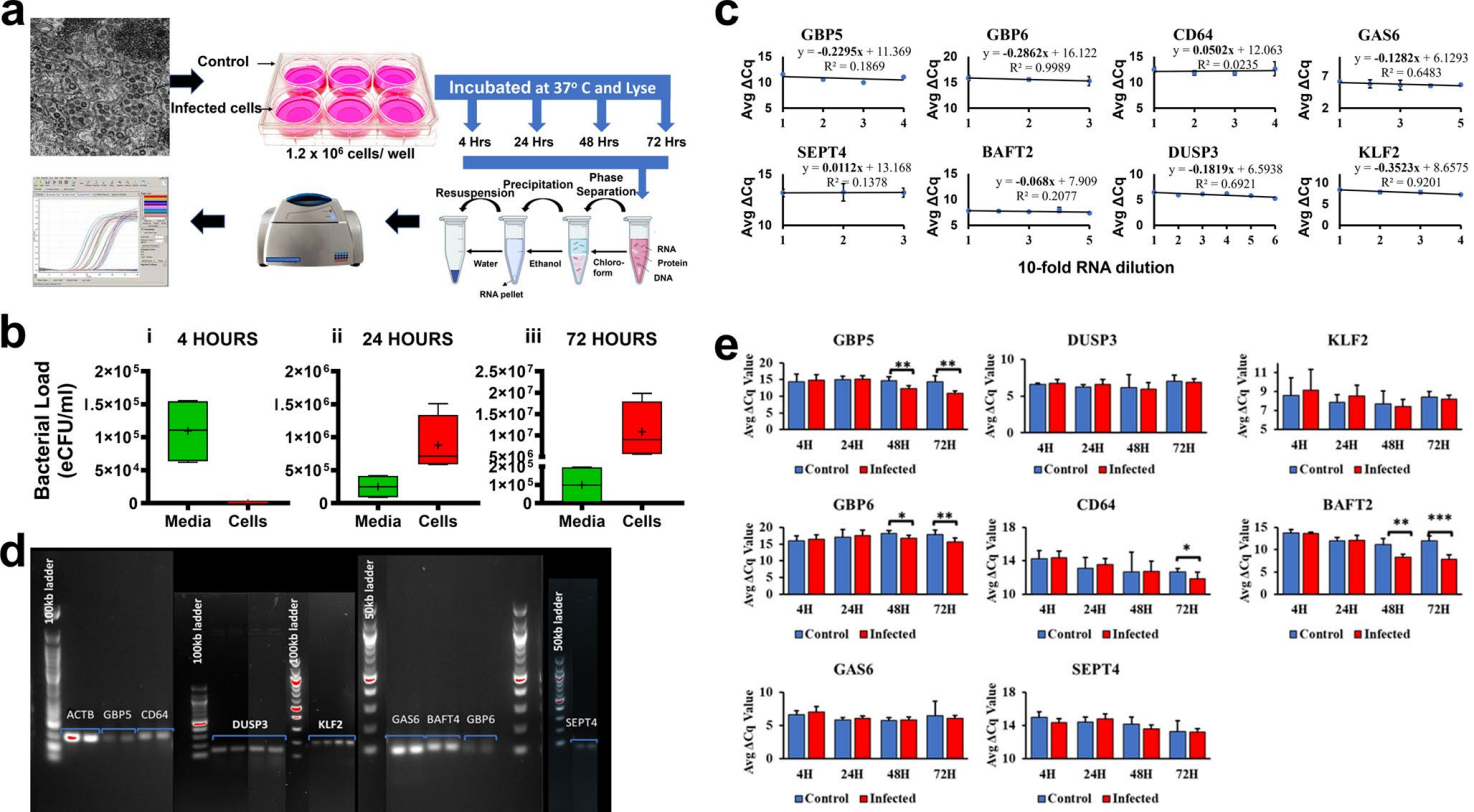
Assessing primer performance in measuring gene expression in a tissue culture model of infection. **a**, Illustration of the lung fibroblast infection assay, extraction procedure and PCR amplification cycle. **b**, BCG association with lung fibroblasts increases with time of incubation. Shown are box plots of bacterial load in eCFU/ml in infected media (green) and in infected lung fibroblast cells (red) after (i) 4 h, (ii) 24 h, and (iii) 72 h of incubation. **c**, Amplification efficiency of the target and reference primers compared. Line graph showing average ΔCq values between target and reference primers at serial dilutions of mRNA. A slope of close to 0 indicated similar amplification efficiency of the target genes and the reference gene ACTB. **d**, Agarose gel electrophoresis for amplified targets. **e**, Upregulated expression of genes in infected human lung fibroblast cells. Bar graphs showing average (Avg.) normalised Cycle quantification (Cq) values as a ratio to Avg. Cq values for the reference gene, ACTB. The lower the Avg. ΔCq value the higher the gene expression. Mann-Whitney test was used to evaluate for statistical differences. Data are reported as mean values from three repeats of each experiment consisting of 2 uninfected and 4 infected wells, * indicates p-value < 0.05, ** indicates a p-value < 0.01 and *** indicates a p-value < 0.001. Error bars are indicative of the standard deviation of the ΔCq values.

The in-vitro experiment performed to induce gene expression in lung fibroblast cells is illustrated in Fig. 3a. MRC-5 cells were successfully infected with BCG bacilli as shown by the progressive shift in the molecular bacterial load (estimated colony forming units per ml (eCFU/ml) from extracellular media to cells from 4 to 72 h of incubation (Fig. 3b). The reference genes *ACTB*, *B2M* and *GAPDH* were initially assessed for invariance in their expression within lung fibroblasts irrespective of experimental conditions and reproducibility between repeats. *ACTB* showed the least variation in its expression for all hours of incubation thus chosen as the appropriate control gene to normalise target expression (Supplementary Fig. 1). B2M expression was highly variable. For validity of data normalisation and calculation of the ΔCq method, primer amplification efficiency of the target genes must be equal to the reference gene^21^ and this was achieved as shown by the slope values in Fig. 3c. Gel electrophoresis of all the RT-qPCR products confirmed there were no primer dimers formed by the designed primers (Fig. 3d**).** Subsequently, the model revealed that expression of HGM *GBP5* (*p* = 0.0080 and *p* = 0.0012), *GBP6* (*p* = 0.0232 and *p* = 0.0042) and *BATF2* (*p* = 0.0024 and *p* = 0.0009) was significantly higher in infected- than uninfected- cells by 48 h and 72 h of incubation, respectively (Fig. 3e). *CD64* achieved differential expression in infected cells by 72 h of incubation (*p* = 0.022), suggesting a delayed response to infection compared to other genes. Expression of the other gene markers, *DUSP3*, *KLF2*, *GAS6* and *SEPT4*, did not differ between infected and uninfected cells at all incubation points. These results indicated that the designed primers could optimally detect targets and were able to show variation in amounts of targets between infected and uninfected cells. The additional gene marker panel described in Fig. 2b and HGM *C1QB* primers were optimised with only in-silico analysis and cell-free medium and not with RNA from in-vitro infection model because they were adopted at the clinical validation stage.

### Assay multiplexing and analysis of precision

**Figure 4:**
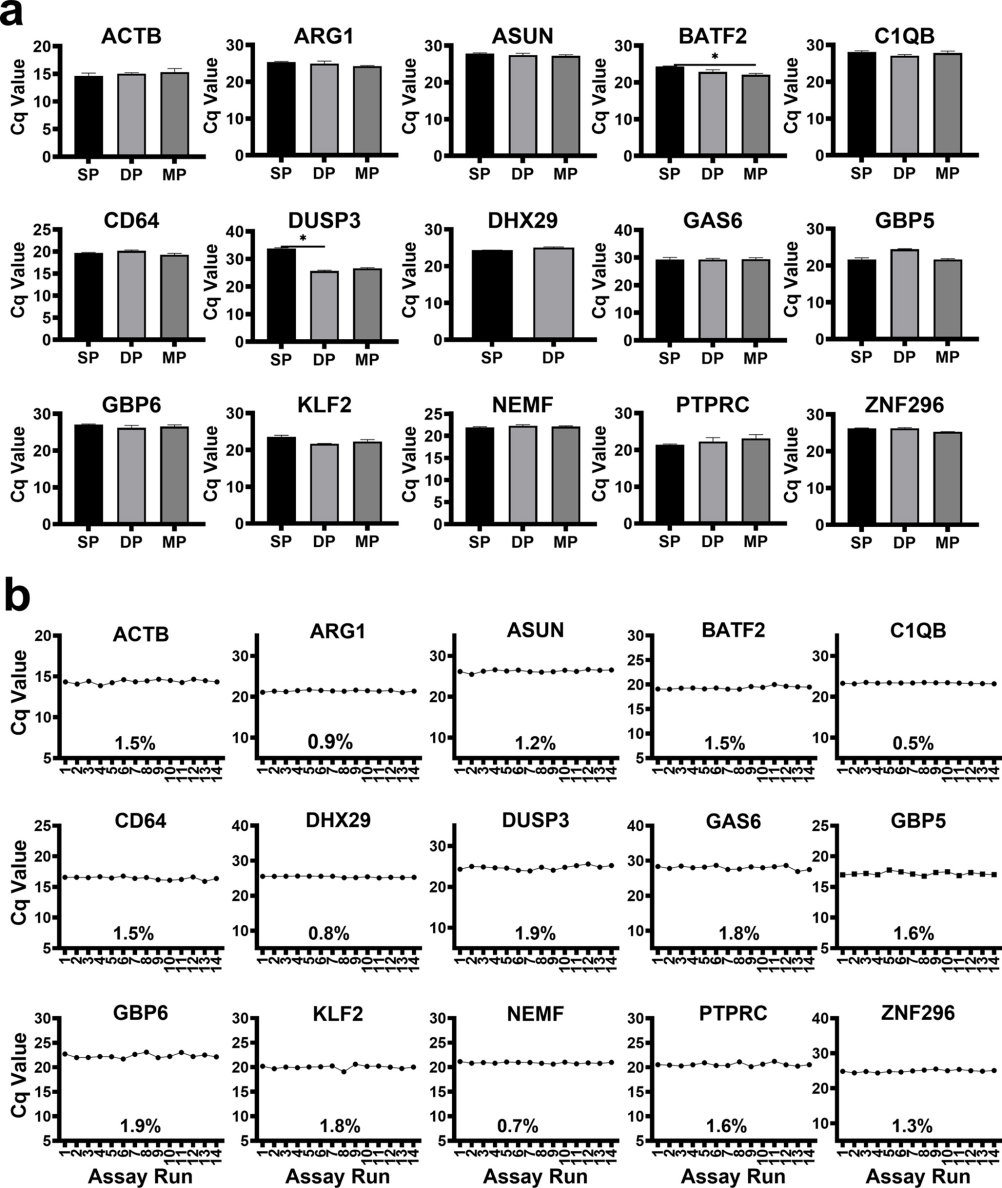
Assay Optimization. Shown are Cq values measured from an ATB participant sample. **a**, Assay multiplexing: SP - Single-target reaction, DP - Double-target reaction and MP - Multiplex, mean expression levels. Comparison was performed using the Kruskal-Wallis test and Dunn’s Test with Bonferroni corrected p-values for multiple comparisons. A p-value of 0.05 or less was considered significant and * is equivalent to *p* < 0.05. **b**, Assay precision testing line graphs showing assay read-out Cq values. Assay technical repeat runs 1–6 were performed on 4th of November 2022 and assay technical repeat runs 7 to 14 were performed on 6th February 2023. Percentage values within the graph represent the Cq CVs between runs for target genes.

For all the gene targets, there was no difference in average Cq value detected in the single-target detection reaction (single plex -SP) assay compared to the three- or four- (multiplex - MP) target detection reaction assay except for *DUSP3* and *BATF2* which had lower Cq values in MP and DP respectively (Fig. 4a). The final multiplex reactions consisted of 5 panels namely: Panel 1: *GBP5*, *KLF2*, *DUSP3* and *ACTB;* Panel 2: *PTPRC*, *BATF2*, *SEPT4* and *GBP6;* Panel 3: *GAS6*, *ASUN*, *NEMF*, and *C1QB;* Panel 4: *ZNF296* and *ARG1*; Panel 5: *CD64* and *DHX29.* Median Cq coefficient of variation (CV) was 1.5% [IQR: 1.1-1.7%] for all gene targets in the multiplex assay indicating adequate precision of the assay even when measured after another freeze thaw cycle (Fig. 4b). Median standard deviation (SD) of these Cq values was 0.27 [range: 0.11–0.48] for all gene target except *SEPT4* [SD = 3.3].

### Assay efficiency, limit of detection (LOD) and limit of quantification (LOQ)

**Figure 5:**
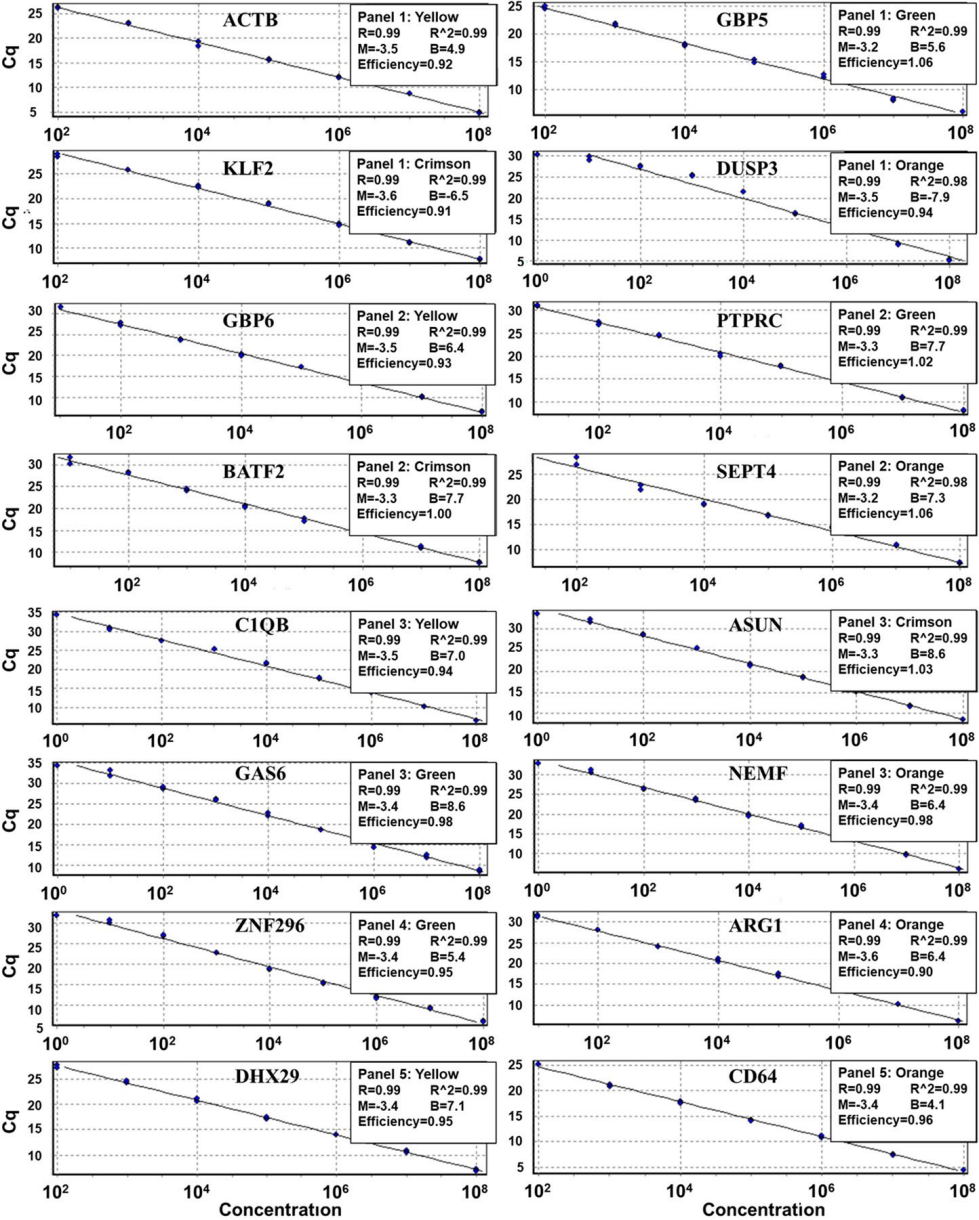
Assay efficiency assessment. PCR efficiency represented by the best of fit regression line of the standard curve for the HGM targets. Cq – quantification cycles, the x-axis shows the concentration of standard target sequences in copies/µl. Blue dots in the curves represents standard target sequencies quantified at different concentrations. Assay threshold value for all targets was set at 0.01 except *GBP6* whose threshold was set at 0.005 because it had low expression in clinical samples. Cycling A - the amplification cycle in the Yellow (HEX), Green (FIM), Crimson (ATTO700) and Orange (ROX) channel. Panel 1/2/3/4/5 are the multiplex reaction panels; Panel 1 to 3 each consisted of 4 HGM and panel 4 and 5 consisted of 2 HGM. R^2 value - coefficient of determination and the R value - Pearson correlation coefficient is the square root of R^2. M and B – represent the slope (M) and the intercept (B) of the standard curve. Efficiency – reaction efficiency by which the independent variable correlates with the dependent variable.

The assay had acceptable dynamic range for target nucleic sequence amplification with linear regression equation Pearson correlation coefficient (R) and coefficient of determination (R^2^) values above 0.98. This indicated that the designed primers and probes reliably detected their specific targets (Fig. 5). The average slope of the standard curve was between the acceptable range of -3.1 and − 3.6. Additionally, all primer sets had good efficiency (90 to 110%) with true statistical doubling for amplification of their specific targets in a multiplex assay (Fig. 5).

LOD was defined as the lowest concentration at which the analyte could be accurately measured in both replicates for a gene marker target with 95% confidence (probability)^30^. All gene targets were accurately detected at a median LOD concentration of 292 copies/µl [IQR: 215-358.3 copies/µl] (Table 2). LOQ was defined as the lowest average concentration of RNA for a gene marker that was accurately measured between 2 replicates with a CV of less than 25%^31^. The median LOQ for all the target standards was 61.7 copies/µl [IQR: 29.4-176.3 copies/µl]. Table 2 shows the LOQs for optimal gene target detection and the corresponding Cq and CV values.

**Table 2:** Assay limit of detection and limit of quantification.

No.	Gene Target	LOD	LOQ		
		Copies/µl	Copies/µl	Cq	CV
1	ACTB	355	28.7	31.0	13.6%
2	ASUN	221	221	31.2	19.8%
3	ARG1	739	29.4	31.4	24.1%
4	BATF2	197	82.1	31.7	20.6%
5	C1QB	382	23.6	34.3	4.8%
6	CD64	178	64.6	28.8	4.4%
7	DHX29	354	29.4	33.6	18.8%
8	DUSP3	159	34.1	32.9	20.6%
9	GAS6	298	40.5	33.1	1.1%
10	GBP5	441	162	29.6	2.6%
11	GBP6	275	59.2	28.7	16.9%
12	KLF2	286	219	31.4	14.2%
13	NEMF	368	244	29.3	7.0%
14	PTPRC	328	23.2	31.0	11.3%
15	SEPT	282	282	31.0	1.8%
16	ZNF296	192	64.2	30.7	1.5%

### Assay quantitative read-out in well characterised participants

A total of 204 participants (61 ATB, 82 ORDs, 24 LTBI and 37 HC) were included in assay clinical evaluation. Supplementary Fig. 2 summaries the procedures involved in the application of the assay to clinical samples. Participant demographic, clinical and laboratory data is shown in Table 3. ATB participants were MGIT culture and Xpert MTB/RIF positive while ORDs participants were MGIT culture and Xpert MTB/RIF negative. LTBI participants were QFT-Plus positive while HC participants were QFT-Plus negative. 38% (31/82) of ORDs participants were also QFT-Plus positive. Extracted median RNA yield was 98.2 ng/µl [IQR: 73.4–130] for ATB, 66.8 ng/µl [IQR: 51.9–92.5] for ORDs, 59 ng/µl [IQR: 44.1–84.6] for LTBI and 91.2 ng/µl [IQR: 72.2–118] for HC participants. RNA yield from ATB participants was significantly more than extracted yield from LTBI and ORDs participants (*p* < 0.001) and RNA yield from LTBI participant was significantly lower than yield for HC participants (*p* = 0.03). No difference in extracted RNA yield was observed between ATB and HC participants.

**Table 3:** Participant demographic and clinical characteristics.

Participant characteristics	ATB [*n* = 61]	ORDs [*n* = 82]	LTBI [*n* = 24]	HCs [*n* = 37]
Sex (male); n [%]	48 [79]	61 [74]	7 [34.7]	19 [52.9]
Age (years); median [IQR]	32 [24–37]	42 [31–52]	36 [30–42]	26 [25–31]
Weight (kgs); median [IQR]	50 [46.5–55]	55.6 [48–61]	62.5 [53–71]	62 [58–69]
Having Cough; n [%]	61 [100]	80 [98]	1 [4]	2 [5]
Night sweats; n [%]	42 [69]	61 [74]	2 [8]	1 [3]
Loss of Weight; n [%]	59 [97]	60 [73]	3 [12.5]	0
Smoking; n [%]	19 [31]	23 [28]	1 [4]	5 [13.5]
Firewood cooking; n [%]	5 [8]	18 [22]	2 [8]	2 [5.4]
Alcohol Use; n [%]	23 [38]	33 [40]	4 [16.7]	10 [27]
BCG Vaccine; n [%]	59 [97]	80 [98]	24 [100]	35[94.6]
Treated for TB before; n [%]	11 [18]	16 [20]	1 [4]	0
Can read; n [%]	54 [89]	76 [93]	23 [95.8]	37 [100]
Employed; n [%]	36 [59]	54 [66]	13 [54.2]	19 [51.4]
Married; n [%]	22 [36]	51 [62]	18 [75]	25 [67.6]
HIV Status; n [%] Positive	22 [36]	37 [45]	5 [20.8]	0
Negative	39 [64]	45 [55]	19 [79.2]	37 [100]
CD4 Count (cells/mm3) median [IQR]	220 [140.5-367.5]	323 [218–423.5]	501 [459–563]	NA
IGRA test; n [%] Positive	49 [80]	31 [38]	24 [100]	0
Negative	3 [5]	44 [54]	0	37 [100]
Indeterminate	9 [15]	7 [9]	0	0
RNA Yield (ng/µl); median [IQR]	98.2 [73.4–130]	66.8 [51.9–92.5]	59 [44 -84.6]	91.2 [72.4–117]
Xpert MTB/RIF; n[%] Positive	61 [100]	0	ND	ND
Negative	0	82 [100]		
MGIT (positive); n[%] Positive	61 [100]	0	ND	ND
Negative	0	82 [100]		

ND = Not done, NA = Not applicable.

**Figure 6:**
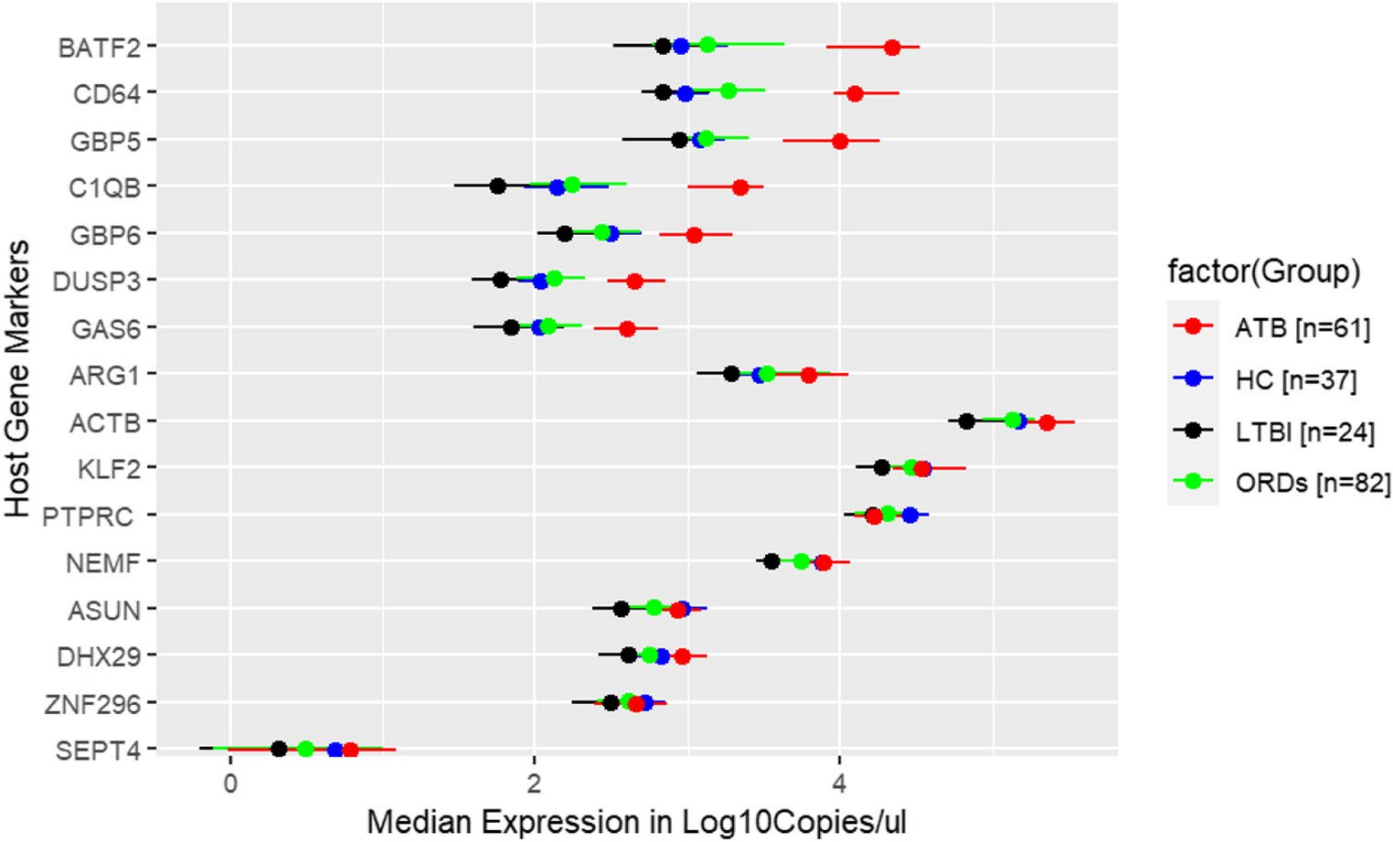
Clinical assay evaluation. Stratified median log_10_copies/µl expression with IQRs of the evaluated HGM.

The host-gene RT-qPCR assay efficiently detected the target markers with minimal dispersion among same group participants. Median expression for ATB was above the assay LOD and LOQ for all gene markers except *SEPT4*. Expression of 7 evaluated gene markers namely *BATF2*, *CD64*, *GBP5*, *C1QB*, *GBP6*, *DUSP3*, and *GAS6* showed distinct upregulation of the genes in ATB compared to either HC, LTBI or ORDs (Fig. 6). Notably, expression levels for LTBI individuals were lower than expression levels of either HC, ORDs, or ATB for all markers, indicating gene marker suppression in LTBI compared to other groups. Table 4 shows the median with IQR of the assay Cq values and the concentration in copies/µl of the measured targets in whole blood.

**Table 4:** Assay quantitative read-out among well characterised groups of participants.

Gene Marker	ATB		ORDS		LTBI		HC	
	Cq	Copies/µl	Cq	Copies/µl	Cq	Copies/µl	Cq	Copies/µl
ACTB	15.9 [15.3–16.8]	226,500 [129000.0–349500.0]	16.7 [16.2–17.4]	135250.0 [86612.5–192250.0]	17.8 [16.9–18.2]	67,050 [51100–120750]	16.5 [16.1–17.0]	152500.0 [116500.0–199000.0]
ARG1	23.1 [22.1–23.9]	6180.0 [3710.0–11400.0]	24.0 [22.5–24.9]	3325.0 [ 1926.3–8793.8]	24.9 [24.2–25.7]	1952.5 [1146.3–2893.8]	24.1 [23.6–24.8]	3265.0 [2050.0–4260.0]
ASUN	26.7 [26.1–27.1]	846.0 [622.5–1242.5]	27.1 [26.6–27.7]	603.8 [419.5–869.3]	27.9 [26.7–28.5]	364.8 [237.4–803.6]	26.6 [26.0–26.8]	902.5 [772.0–1390.0]
BATF2	21.3 [20.7–22.8]	21950.0 [8210.0–33850.0]	25.4 [23.7–26.6]	1335.0 [585.0–4340.0]	26.4 [25.2–27.4]	688.3 [321.1–1525.0]	26.0 [24.9–27.0]	886.8 [439.1–1778.8]
C1QB	24.7 [24.2–25.9]	2190.0 [992.5–3190.0]	28.5 [27.3–29.5]	175.0 [94.0–396.3]	30.3 [28.8–31.2]	56.0 [29.3–144]	28.8 [27.6–29.5]	149.5 [91.9–325.0]
CD64	19.0 [18.0–19.5]	12700.0 [9020.0–24600.0]	21.9 [21.0–23.0]	1842.5 [871.8–3203.8]	23.3 [22.3–23.8]	691.8 [502.6–1402.5]	22.9 [22.3–23.2]	949.5 [774.0–1355.0]
DHX29	26.0 [25.4–26.4]	918.0 [694.0–1355.0]	26.7 [26.3–27.4]	560.0 [360.9–760.9]	27.2 [26.4–27.9]	407.8 [263.5–706.6]	26.4 [26.0–27.1]	719.0 [448.0–898.0]
DUSP3	26.1 [25.4–26.7]	442.5 [294.0–730.5]	28.0 [27.2–28.8]	133.3 [75.3–215.3]	29.2 [28.7–29.8]	58.7 [38.9–83.2]	28.2 [27.8–28.6]	110.5 [86.5–151.5]
GAS6	28.3 [27.6–29.1]	394.5 [245.0 -652.0]	30.2 [29.3–30.9]	119.9 [78.0–204.0]	30.9 [29.8–31.7]	69.4 [40.1–154.9]	30.3 [29.8–30.8]	108.8 [76.8–153.9]
GBP5	19.7 [18.9–20.9]	9920 [4255.0–18150.0]	22.6 [21.7–23.2]	1312.5 [825.0–2493.8]	23.1 [22.9–24.3]	878 [371.3–1052]	22.7 [22.2–23.0]	1195.5 [928.5–1635.0]
GBP6	25.4 [24.5–26.3]	1085.0 [656.5–1990.0]	27.5 [26.7–28.5]	276.8 [144.7–484.6]	28.4 [27.2–29.1]	155.5 [104.8–372.4]	27.4 [26.5–27.8]	332.0 [239.0–533.5]
KLF2	21.7 [20.7–22.4]	34350.0 [22500.0–67700.0]	21.9 [21.5–22.6]	29,500 [19387.5–38175.0]	22.7 [21.8–23.2]	18,625 [12907.5–32112.5]	21.7 [21.3–22.1]	35650.0 [26050.0–44250.0]
NEMF	21.9 [21.2–22.2]	7710.0 [5950.0–11750.0]	22.4 [21.9–22.9]	5537.5 [3765.0–7436.3]	23.0 [22.2–23.4]	3577.5 [2806.3–6186.3]	21.8 [21.1–22.3]	7780.0 [5650.0–12650.0]
PTPRC	21.6 [20.9–22.0]	16600.0 [12600.0–28450.0]	21.3 [20.8–22.0]	20375.0 [12500.0–30037.5]	21.7 [21.2–22.2]	16,350 [10830–22600]	20.8 [20.4–21.4]	30100.0 [19000.0–38450.0]
SEPT4	33.2 [31.9–36.0]	6.0 [1.0–12.4]	34.8 [32.9–35.9]	3.1 [0.8–10.0]	34.3 [33.3–35.9]	2.1 [0.6–4.9]	33.3 [32.3–35.0]	5.0 [1.7–8.9]
ZNF296	25.4 [24.7–26.3]	460.0 [247.0–740.0]	25.6 [25.1–26.3]	411.0 [255.5–565.6]	26.0 [25.5–26.9]	310.5 [172.5–416.0]	25.2 [24.7–25.5]	535.0 [423.0–709.0]

*All data is reported as median quantified level with IQR for each gene marker within each characterized group of participants.

**Figure 7:**
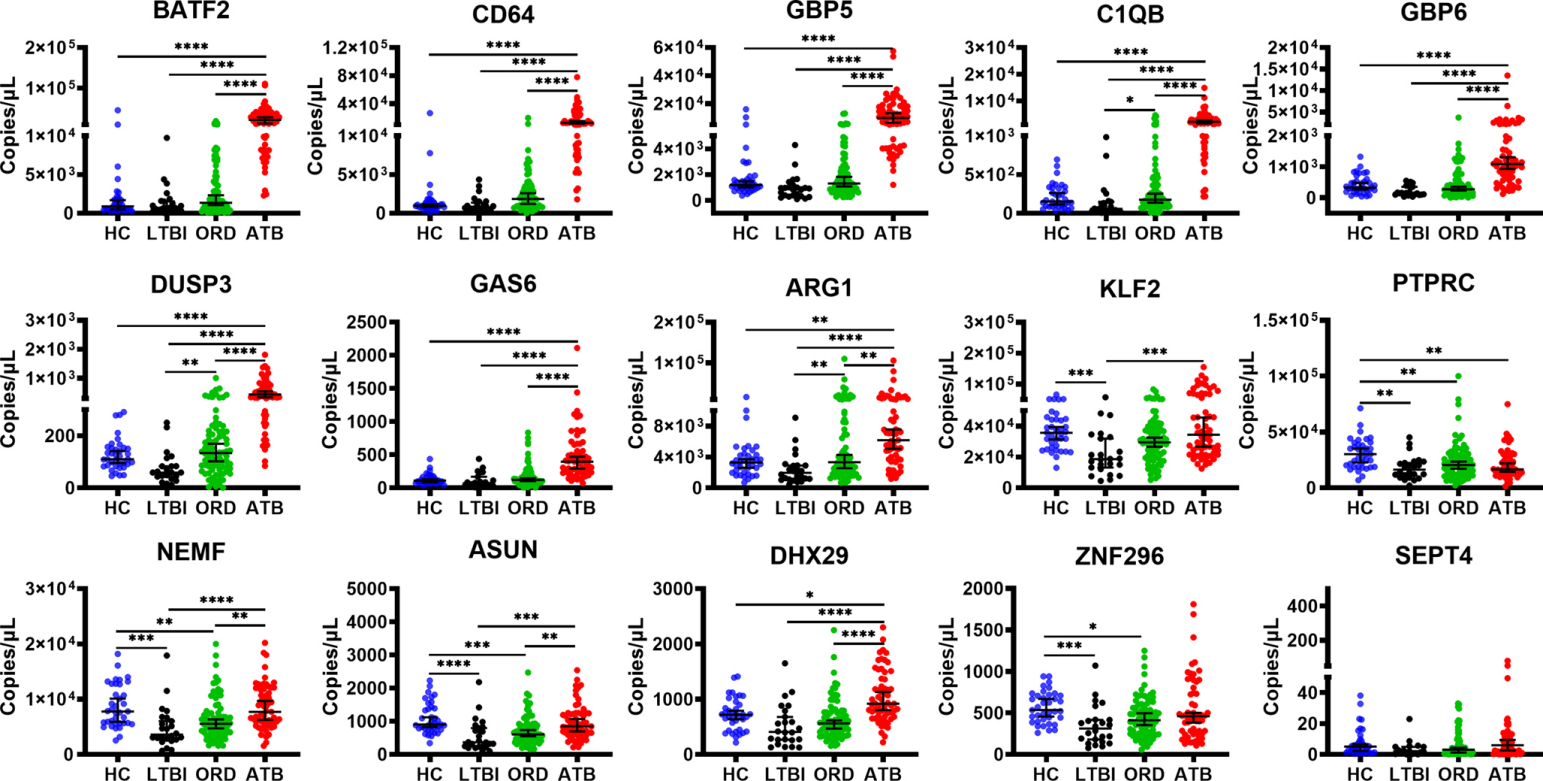
Host-gene marker expression levels among the study participants. Shown are scatter plots of measured expression levels among the participants namely HC (*n* = 37), LTBI (*n* = 24), ORD (*n* = 82), ATB (*n* = 61). Lines show the median and 95% confidence intervals. Statistical comparison was performed using the Kruskal-Wallis test and Dunn’s Test with Bonferroni corrected p-values for multiple comparisons. *=<0.05, **=<0.01, ***=<0.001, and ****=<0.0001.

When the absolute measurements of the gene levels were compared, HGM expression of *BATF2*, *CD64*, *GBP5*, *C1QB*, *GBP6*, *DUSP3*, *GAS6*,* ARG1*, and *DHX29* was significantly upregulated in ATB individuals compared to expression in ORDs, LTBI or HC (Fig. 7). Whilst expression levels of HGM *KLF2*, *PTPRC*, *NEMF*, *ASUN*, and *ZNF296* were significantly downregulated in LTBI compared to HC. Notably, expression levels of *KLF2*, *NEMF*, *ASUN*, and *ZNF296* were akin in ATB and HC participants.

## Discussion

The aim of this work was to identify, design, optimize and clinically evaluate a host gene-based RT-qPCR assay for TB diagnosis. A total of 16 gene targets were selected and converted into 5 multiplex RT-qPCR panels of two to four detection targets per panel and evaluated for best accuracy to diagnose ATB, LTBI and distinguish them from ORDs and HC. While other studies attempted the RT-qPCR approach using SYBR green and single targets per reaction^12,14,15,34,35^, our RT-qPCR integrates dual-labelled hydrolysis probes to detect multiple targets in a single reaction minimising sample wastage, and reducing labour and material costs^36^. Furthermore, the SYBR green dye binds all double stranded DNA and is thus reported nonspecific, generating false positive signals^36^. In contrast, we have shown that our assay is specific and distinguishes ATB from LTBI, ORDs and HC individuals in a near point-of-care (POC) context.

In an in-vitro infection model, the designed primers detected and amplified HGM *GBP5*, *DUSP3*, *KLF2*, *GBP6*, *BATF2*, *CD64*, *SEPT4*, and *GAS6* in both uninfected and infected lung fibroblast cells. Differential expression between infected and uninfected lung fibroblasts was observed at 48 h and/or 72 h for *GBP5*, *GBP6*, *BATF2* and *CD64* genes an indication that the designed primers could optimally measure variation in amounts of expressed gene targets between infected and uninfected cells. Absence of activated gene expression at earlier times (4 h and 24 h) may signify the slow rate of endocytosis, hence delaying the signal transduction to induce gene expression. In the same context, it could be that genes *DUSP3*, *KLF2*, *SEPT4*, and *GAS6*needed longer time of pathogen exposure to exhibit differential gene expression. In-vivo, lung fibroblasts are constituent cells of the TB granuloma, which make them ideal in-vitro tissue culture model of infection for both MTB and BCG^37,38^. Furthermore, ex-vivo analysis of fibroblasts from normal lung tissue of LTBI individuals demonstrated that these cells were infected with MTB in-vivo^39^.

The multiplex RT-qPCR assay demonstrated excellent performance for target specific amplification with high efficiency and large dynamic range with true statistical doubling in each cycle. This indicated that optimal primer and probe pairs were designed and that reaction conditions were well optimized which ensured accurate detection of the targets^30^. The assay also displayed high precision, indicating consistent results with minimal variability. Consequently, the assay LOD and LOQ were determined demonstrating the assay’s ability to quantify low concentration target genes in a multiplex reaction^36^. All this indicated that the detection signals of the assay were within the linear range making the assay robust, highly precise, and specific.

Clinical evaluation of the assay in a group of well-characterized clinical participants demonstrated clear differential expression of the genes among different groups supporting its potential for diagnostic application. All gene targets were detected below the LOQ Cq value with minimal dispersion among participants within the same group except for SEPT4. The absolute method used to quantify expression profiles is accurate since it accounts for the amplicon length^36^. Other researchers have also found the RT-qPCR assay a valuable tool for accurate quantification of transcriptional markers in whole blood for TB diagnosis although the quantification levels were rarely reported^12–14^. Assay quantitative read-outs are important for comparability and diagnostic cut-off determination which is crucial for sensitivity and assessing response to treatment in the context of managing ATB.

Our results are comparable with previous reports of upregulated expression of genes in ATB participants compared to ORDs, LTBI or HC^12,14,33,40^ and HGM suppression in LTBI compared ATB^4,15^. This observed suppressed expression profile in LTBI compared to HC individuals has been fully deciphered and reported by our team elsewhere^25^. Whilst a detailed evaluation of the expression profiles among ATB participants compared to ORDs participants and how the profiles in ATB change with treatment is being undertaken and will be reported separately. The clinical significance of the expressed expression profiles in ATB compared to ORDs with robust modelling to identify models highly discriminative of the two conditions will be included in that report. Nonetheless, the results reported here show that LTBI is associated with a suppressed expression profile while ATB leads to an upregulation of HGM an indication that different gene markers maybe required for diagnosis of these two physiologically distinct states of TB disease. Additionally, immune regulatory genes namely *KLF2*, NEMF, *ASUN*, and *ZNF296*showed comparable expression in HC and ATB participants. Others have also reported similar expression levels of some of these HGM between HC and ATB individuals^15^. This implies that MTB is may not necessarily be subverting host regulatory system in ATB as seen in LTBI. Future research studies to understand the normal level of expression exhibited by these immune regulators in ATB acute immune activation are warranted.

SEPT4 was reported as part of the Bloom144^10^, and Kaforou44^4^ signatures for distinguishing ATB from ORDs, the Zak 16^9^ and RISK4^41^ signatures for ATB progression from LTBI and the Dawany251^42^ signature for TB detection among HIV/TB infected and HIV mono-infected participants, although its quantitative read-outs were never reported. This SEPT4 performance was not replicated in our study. Nevertheless, Since this target standard region was adequately amplified with good efficiency, the designed primer-probe pairs were optimal for its quantification and the possibility of this gene expression level being low in participant samples was more likely. Darboe et al. also reported poor reproducibility with high redundant signal yield for SEPT4 in a microfluidic RT-qPCR assay^16^ which further indicates low levels of target gene in clinical samples for RT-qPCR measurements. This illustrates the need to translate microarray measured findings of DE genes into simple near POC test like the RT-qPCR to confirm reproducibility.

Composite biological functions of the evaluated HGM include immune response signalling and regulation^43,44^, defence mechanism against pathogens^45–47^, and biological interaction between organisms^48^, transcription initiators^49^, translation initiators^50^, ribosomal quality control gene^51^, and lastly genes involved in cell cycle mitosis^52^, proliferation^53,54^, and differentiation regulators^55^ (refer to Supplementary Table 2). These results point to the need to translate microarray measured findings into simple near POC test like the RT-qPCR to confirm reproducibility. Combining RT-qPCR with Whole Genome Sequencing (WGS) to confirm size of the targets in participant samples after RNA extraction might also be beneficial.

We acknowledge limitations of this work which include use of BCG- an attenuated form of MTB complex to induce gene expression. BCG induced gene expression may not be the same as that induced by wildtype MTB, and in-vitro host-pathogen interaction may not recapitulate the in-vivo situation. However, this step was intended to verify whether the designed primers and probes could detect and amplify the target regions with differential HGM quantification between differing experimental conditions. Secondly, underlying cause of disease among ORDs participants was never confirmed. Clinical evaluation of the assay was performed in the same local Blantyre region and study findings may lack generalizability although the evaluated gene targets were discovered in different geographical regions and those frequently reported in different signatures were chosen. Also, LTBI and HC sample sizes were small, and this may impact statistical power of the analysis. Lastly, while our HGM panel targets several key genes associated with LTBI and ATB, there may be other genes that are also relevant to TB diagnosis not included. Nonetheless, this assay can easily be adopted for newly identified HGM thus continuous updating and refining of gene marker selection based on emerging research and clinical findings will provide a more comprehensive assay.

In conclusion, our study has provided a method to make HGM-based diagnosis of LTBI and ATB forms of TB at near point-of-care. This method is robust, highly sensitive and specific for gene expression quantification enabling prompt participant clinical classification. Further studies are needed to ascertain robustness and reproducibility of the gene marker expression in different genetic backgrounds.

## Electronic supplementary material

Below is the link to the electronic supplementary material.

Supplementary Material 1

## Data Availability

All data generated and analysed during this study are available on the University of St Andrews OneDrive and is accessible upon request and meeting ethical requirements as per study participants consent from Dr Wilber Sabiiti ws31@st-andrews.ac.uk.
