# Supplementary material for: Specific human gene expression in response to infection is an effective marker for diagnosis of latent and active tuberculosis
Source: Sci Rep. 2024 Nov 6;14:26884. doi: 10.1038/s41598-024-77164-5 (PMC11541504; doi:10.1038/s41598-024-77164-5)
Supplement: Supplementary file 1 — Supplementary Material 1 [file 41598_2024_77164_MOESM1_ESM.docx]

**Specific human gene expression in response to infection is an effective marker for diagnosis of latent and active tuberculosis**

Ritah Nakiboneka^1,2,3,4^; Natasha Walbaum^1^; Emmanuel Musisi^1,5^; Michael Nevels^6^; Tonney Nyirenda^2^; Marriott Nliwasa^2,3^; Chisomo L. Msefula^2,3,4^; Derek Sloan^1^; Wilber Sabiiti^1^.

***Supplementary Table 1: Published literature citing plausible HGM***

| No. | Signature Name | No of genes | Genes in signature | Paper citation |
| --- | --- | --- | --- | --- |
| 1 | Anderson42 - Distinguished TB from LTBI | 42 | ACTA2, ALKBH7, APOL6, C11ORF2, C20ORF201, C21ORF57, C8ORF55, CARD16, CLIP1, CRIP2, DEFA1, DEFA1B, DEFA3, DGCR6, DNAJC30, E4F1, FBLN5, GBP5, GBP6, GNG3, HS.538100, IMPDH2, KLHL28, LCMT1, LGTN, LOC389816, LOC400759, LRRN3, MFGE8, NDRG2, NME3, NOG, PAQR7, PASK, PASK, PHF17, RAP1A, SIVA, SNHG7, TGIF1, U2AF1L4, UBA52 | Anderson et al. 2014; PMID: 24785206 |
| 2 | Anderson51 - Distinguished TB from other diseases | 51 | ALAS2, ALDH1A1, C1QB, C20ORF103, C3HC4, CAST, CCDC52, CD226, CD79A, CDKN1C, CEACAM1, CYB561_variant 1, CYB561_varient 3, DEFA1, F2RL1, FER1L3, FRMD3, GBP3, GBP5, GBP6,GRAMD1B, HLA-DRB1, HLA-DRB5, HLA-DRB6, HPSE, HS.106234, HS.171481, JUP, KCNJ15, KIFC3, KLHDC8B, KREMEN1, LOC389386, LOC389386, LOC642678, LOC647460, LOC653778, MIR1974, NCF1B, OSBPL10, PDCD1LG2, SCGB3A1Y, SEMA6B, SIGLEC14, SMARCD3, SNORD8, TNFRSF17, TPST1, VAMP5, ZBED2 | Anderson et al. 2014; PMID: 24785206 |
| 3 | BATF2 - Discriminates Active TB from healthy individuals | 1 | BATF2 | Roe et al., 2016; PMID: 27734027 |
| 4 | Berry et al - Discriminates Active TB from other inflammatory and infectious diseases | 86 | 86-genes - too numerous to list | Berry et al., 2010; PMID: 20725040 |
| 5 | Bloom et al., - Signature distinguishes TB from other lung infections | 144 | 144- genes --too numerous to list | Bloom et al., 2013; PMID: pone.0070630 |
| 6 | Cai et al., - Complement Marker for Active TB | 3 | C1qA, C1qB, C1qC | Cai et al.,2014; PMID: 24647646 |
| 7 | Dawany et al - Detects TB in HIV positive and negative patients | 251 | 251 genes -too numerous to list | Dawany et al. 2014; PMID: 24587128 |
| 8 | Duffy10 - Discriminates ATB from other diseases | 10 | CD160, LAG3, CD36, FCGR1B, CERKL, GBP6, ID3, C1QB, CD40LG, ZDHHC19 | Duffy et al., 2019; PMID: pone.0219322 |
| 9 | Gjoen7 - TB diagnostic signature in children | 7 | MMP9, CD3E, NOD2, GBP5, IFITM1/3, KIF1B, TNIP1 | Gjoen et al., 2017; PMID: s41598-017-05057-x |
| 10 | Gliddon3 - Differentiated ATB from LTBI | 3 | FCGR1A, ZNF296, C1QB | Gliddon et al., 2019; PMID: 10.1101/583674 |
| 11 | Gliddon4 - Distinguished TB patients from other diseases | 4 | GBP6, TMCC1, PRDM1, ARG1 | Gliddon et al., 2019; PMID: 10.1101/583674 |
| 12 | Kaforou27 - Detects TB from LTBI in HIV-Infected and uninfected adults | 27 | GAS6, ANKRD22, LHFPL2, FCGR1A, GNG7, C5, C1QC, FLVCR2, CD79A, VAMP5, C4ORF18, FCGR1B, FAM20A, ZNF296, MPO, GBP6, CXCR5, GAS6, LOC728744, FCGR1C, CCR6, C1QB, SMARCD3, S100A8, CD79B, DUSP3, FCGR1B | Kaforou et al., 2013; PMID: pmed. 1001538 |
| 13 | Kaforou44 - For distinguishing TB from other diseases | 44 | CYB561, LOC196752, HM13, LHFPL2, PPPDE2, RBM12B, PRDM1, CASC1, CYB561, CALML4, HLA-DPB1, ALDH1A1, EBF1, AAK1, PGA5, RNF19A, HS.131087, SERPING1, MIR1974, IMPA2, GJA9, ORM1, MAP7, BTN3A1, PDK4, RP5-1022P6.2, GBP6, UGP2, CERKL, CREB5, CD74, LOC389386, VPREB3, SEPT4, HS.162734, ARG1, MAK, MAP7, C19ORF12, ALDH1A1, DUSP3, LOC100133800, TMCC1, HM13 | Kaforou et al., 2013; PM1D: pmed. 1001538 |
| 14 | Laux da Costa et al. - discriminates between TB and other pulmonary diseases | 3 | GBP5, CD64, GZMA | Laux da Costa et al., 2015; PMID: 26025597 |
| 15 | Maertzdorf4 - TB diagnosis signature | 4 | GBP1, ID3, IFITM3, P2RY14 | Maertzdorf et al, 2015; PMID: 26682570 |
| 16 | RISK4 - Predicts TB progression | 4 | GAS6, SEPT4, CD1C, BLK | Suliman et al., 2018; PMID: 29624071 |
| 17 | Roe3 - TB Progression signature | 3 | BATF2, GBP5, SCARF3 | Roe et al., 2020; PIMD: article/70/5/731/5421263 |
| 18 | Suliman2 - Predicts TB progression | 2 | C1QC, TRAV27 | Suliman et al., 2018; PMID: 29624071 |
| 19 | Sweeney3 - For TB diagnosis and treatment response monitoring | 3 | GBP5, DUSP3, KLF2 | Sweeney et al., 2016; PMID: 26907218. |
| 20 | Zak16 - TB risk signature; detects progression from LTBI to ATB | 16 | ETV7, FCGR1A, FCGR1B, GBP1, GBP2, GBP5, SCARF1, SERPING1, STAT1, TAP1, TRAFD1, ANKRD22, APOL1, BATF2, GBP4, SEPT4 | Zak et al., 2016; PMID: 27017310 |

***Supplementary Table 2: Summarised biological functions of the selected genes***

| Gene | Biological function |
| --- | --- |
| GBP5 | Induce by IFN-γ^1,2^ |
|  | Plays a role in innate immunity and inflammation^3^: |
|  | - Cytokine signalling in the innate immune system |
|  | - IFN-γ signalling in the adaptive immune system |
|  | - Activates the NLRP3 inflammasome assembly |
| GBP6 | Induced by IFN-γ^2^ and offers protection from several pathogens |
|  | Downregulation associated with poor cell differentiation and prognosis in cancers^4^ |
| DUSP3 | Inactivates target kinases and regulates mitogen-activated protein (MAP) kinase members^5^ |
|  | MAP kinase members are associated with cellular proliferation and differentiation |
|  | Involved in cellular proliferation and differentiation regulation |
| BATF2 | Basic leucine zipper transcription factor 2 participates in the immune system through controlling differentiation of lineage specific cells^6^ |
|  | Predominantly expressed in monocytes and macrophages. |
|  | Vital in macrophage activation in Mycobacterial infections. |
| CD64 | Important in both innate and adaptive immune responses. |
|  | Encodes a high-affinity Fc-gamma receptor protein that binds IgG with high affinity^7^. |
|  | Mediates IgG effector functions on monocytes triggering antibody-dependent cellular cytotoxicity (ADCC) of virus-infected cells. |
|  | CD64 is highly expressed on resting monocytes, macrophages^8^, and neutrophils^9^. |
|  | Involved in IFN-γ signalling pathway, is induced by IFN-γ^10^ |
| KLF2 | Krüppel-like Factor 2 (KLF2) - Expressed in the lungs, erythroid and lymphoid tissues^11^ |
|  | Regulator of several inflammatory genes and cytokines by regulating the transcriptional activity of NF-κB through competitive interaction with PCAF^12^ |
|  | GBP5 and DUSP3 inflammatory genes whose activation is through the NF-KB pathway are hence regulated by KLF2. |
|  | Expressed highly in both naïve and memory T cells and monocytes^12^ |
|  | Maintains the viability of T cells in the peripheral lymphoid organs and blood but it also mediates anti apoptotic stage of mature T cell. |
|  | Controls T cell trafficking from the thymus to secondary lymphoid tissues by directly regulating CD62L. |
|  | Highly expressed in mature B cells. its deficiency has been reported to result into immature B cells |
| GAS6 | Encodes a protein called Growth Arrest specific 6 |
|  | Originally found as a gene upregulated by growth arrested fibroblasts. |
|  | Involved in stimulation of cell proliferation |
|  | Frequently overexpressed in many cancers and has been implicated as an adverse prognostic marker^13^ |
|  | Plays a role in viral cell entry by apoptotic mimicry^14,15^ |
| SEPT4 | Member of the Septin gene family and is localised at the mitochondrion^16^ |
|  | Reported to aggravate hypoxia-induced cardiomyocytes apoptosis ^16^. |
| C1QB | Complement C1q B Chain is involved in innate adaptive immunity |
|  | Activation of the classical complement system |
|  | The complement cascade is part of the immune system and enhances the ability of phagocytic cells and antibiotics to kill microbes, clear damaged cells, and induce inflammation^17^. |
|  | C1QB proteins are involved in T cell activation following antigen presentation. |
| ASUN | Involved in the adaptive immune system |
|  | Is a cell cycle regulator |
|  | Acts in the nucleus in connection with other integrator components to mediate recruitment of dynein to the nucleus envelop^18^ |
|  | Involved in centrosome localisation, mitotic spindle organisation |
|  | Protein localisation to the nuclear envelop |
|  | Regulates fertilization, regulates mitotic cell cycle |
| NEMF | Nuclear export mediator factor NEMF; |
|  | Part of the ribosome quality control complex (RQC), a ribosome-associated complex that mediates ubiquitination and extraction of incompletely synthesized nascent chains for proteasomal degradation^19^. |
|  | Responsible for selective recognition of stalled 60S subunits by recognizing an exposed, nascent chain-conjugated tRNA moiety^19^. |
|  | Important for the stable association of LTN1 to the complex. May indirectly play a role in nuclear export (1076 aa)^20^ |
| DHX29 | Encoded protein functions in translation initiation^21^ |
|  | Specifically required for ribosomal scanning across stable mRNA during initiation codon selection^22^ |
|  | Sensing virally derived cytosolic nucleic acid |
|  | Knockout of gene reduces protein translation and impaired proliferation of cancer cells |
| PTPRC | Also known as CD45 |
|  | PTPs are known to be signalling molecules that regulate a variety of cellular processes including cell growth, differentiation, mitosis, and oncogenic transformation^23^ |
|  | CD45 shown to be an essential regulator of T- and B-cell antigen receptor signalling^24^ |
|  | Upregulated expression of PTPRC was associated with poor prognosis in acute myeloid leukemia^25^ |
| ZNF296 | Encodes the zinc finger protein 296 |
|  | Predicted to be involved in positive regulation of transcription by RNA Polymerase II and spermatogenesis^26^ |
|  | Required for proper germ-cell development and embryonic growth^26^ |
|  | Enables sequence specific double stranded DNA binding |
| ARG1 | The ARG1 gene provides instructions for producing the enzyme arginase. |
|  | Arginase enzyme participates in the urea cycle, a series of reactions that occurs in liver cells. |
|  | Increased expression of ARG1 induced by MTB reduces the production of nitric oxide required for MTB killing^27^ |
|  | Expressed in increased amounts in the granuloma of TB patients^28^ |

***Supplementary Figure 1: Showing reference gene expression in MRC-5 cells***


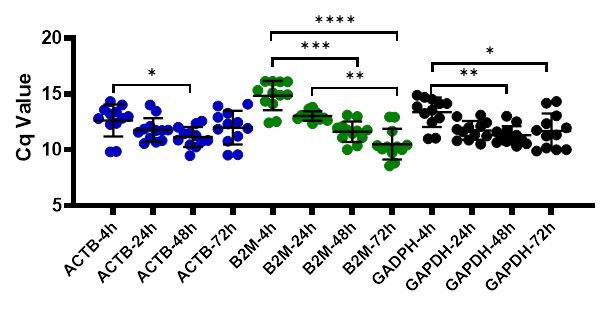


Expression of reference genes in human lung fibroblast cells at the different hours of incubation were expression for ACTB are blue dot, B2M- green dots and GAPDH- black dots. Statistical comparison was performed using Kruskal-Wallis test and Dunn's Test with Bonferroni corrected p-values for multiple comparisons. * Denotes p<0.05, ** denotes p<0.01, *** denotes p<0.001 and **** denotes p<0.0001.

***Supplementary Table 3: Host-gene primers used in the RT-qPCR analysed for hairpin and dimer formation.***

| Oligo Name |  | Length (Mer) | GC Content (%) | Melting Temp (^o^C) | Hairpin (HP) highest HP Tm | Delta G (ΔG) | Heterodimer formation | Amplicon length |
| --- | --- | --- | --- | --- | --- | --- | --- | --- |
| GBP5 | Forward | 21 | 42.9 | 61.7 | 37.5 | -3.14 | -4.41 | 150 |
|  | Reverse | 20 | 45.0 | 61.3 | 23.2 | -3.61 |  |  |
| GBP6 | Forward | 22 | 40.9 | 62 | 25.3 | -3.17 | -3.54 | 107 |
|  | Reverse | 22 | 45.5 | 62 | 31.3 | -3.14 |  |  |
| C1QB | Forward | 20 | 50 | 54.8 | 17.1 | -3.14 | -3.55 | 91 |
|  | Reverse | 23 | 43.5 | 54.2 | 44 | -5.36 |  |  |
| CD64 | Forward | 22 | 45.5 | 64.5 | 36.8 | -6.34 | -3.55 | 158 |
|  | Reverse | 22 | 50.0 | 64.9 | 8.5 | -3.61 |  |  |
| SEPT4 | Forward | 20 | 50 | 62 | 32 | -3.61 | -6.62 | 118 |
|  | Reverse | 19 | 52.6 | 62 | 30.3 | -6.34 |  |  |
| GAS6 | Forward | 21 | 47.6 | 62 | 24.8 | -3.3 | -5.13 | 106 |
|  | Reverse | 22 | 50 | 62 | 36.3 | -4.62 |  |  |
| BAFT2 | Forward | 21 | 47.6 | 62 | 33.4 | -3.55 | -6.14 | 113 |
|  | Reverse | 22 | 50 | 62 | 39.5 | -4.67 |  |  |
| DUSP3 | Forward | 20 | 47.6 | 65 | 51.8 | -5.84 | -7.81 | 178 |
|  | Reverse | 20 | 55.0 | 65.5 | 41.3 | -3.61 |  |  |
| KLF2 | Forward | 21 | 55.0 | 66.2 | 38 | -5.09 | -8.7 | 150 |
|  | Reverse | 20 | 55.0 | 65 | 43.1 | -3.61 |  |  |
| ASUN | Forward | 22 | 61.9 | 50 | 36.1 | -5.38 | -5.38 | 109 |
|  | Reverse | 22 | 61.8 | 45.5 | 46 | -5.38 |  |  |
| NEMF | Forward | 21 | 61.9 | 47.6 | 34.3 | -3.14 | -6.97 | 100 |
|  | Reverse | 19 | 61.9 | 47.4 | 47.8 | -7.05 |  |  |
| DHX29 | Forward | 21 | 62.2 | 47.6 | -5.13 | -5.13 | -3.61 | 94 |
|  | Reverse | 20 | 62 | 50 | 34.4 | -9.75 |  |  |
| PTPRC | Forward | 23 | 62 | 43.5 | 38.5 | -5.84 | -3.3 | 110 |
|  | Reverse | 21 | 61.9 | 52.4 | 23 | -3.14 |  |  |
| ZNF296 | Forward | 20 | 50 | 62.3 | 13.5 | -3.61 | -6.37 | 142 |
|  | Reverse | 20 | 45 | 62.5 | 23.9 | -3.14 |  |  |
| ARG1 | Forward | 22 | 50 | 55.1 | 35.5 | -3.17 | -7.81 | 115 |
|  | Reverse | 22 | 45.5 | 62.2 | 35.1 | -3.9 |  |  |
| ACTB | Forward | 18 | 66.7 | 64.5 | 54.1 | -9.89 | -5.09 | 150 |
|  | Reverse | 24 | 45.8 | 61 | 30.5 | -3.14 |  |  |
| B2M | Forward | 22 | 40.9 | 62 | 28.6 | -5.38 | -6.6 | 116 |
|  | Reverse | 25 | 40 | 62 | 46.2 | -6.97 |  |  |
| GAPDH | Forward | 23 | 43.5 | 62 | 55.8 | -5.38 | -5.12 | 123 |
|  | Reverse | 22 | 50 | 62 | 12.4 | -3.61 |  |  |

***Supplementary Table 4: Designed probe properties***

| No. | Gene Target | Colour | Quencher | Length (bp) | GC Content (%) | Melting Temp (^o^C) | Weight (g/mol) |
| --- | --- | --- | --- | --- | --- | --- | --- |
| 1 | GBP5 | FAM | BHQ1 | 24 | 45.8 | 61 | 8428.7 |
| 2 | GBP6 | HEX | BHQ2 | 24 | 50 | 62.7 | 8733.5 |
| 3 | C1QB | HEX | BHQ1 | 24 | 50 | 62.7 | 8669.5 |
| 4 | CD64 | ROX | BHQ2 | 24 | 54.2 | 64.4 | 8590 |
| 5 | SEPT4 | ROX | BHQ2 | 24 | 54.2 | 64.4 | 8759.1 |
| 6 | GAS6 | FAM | BHQ1 | 25 | 48 | 63 | 8856.1 |
| 7 | BAFT2 | ATTO700 | BHQ1 | 24 | 54.2 | 64.4 | 8614.3 |
| 8 | DUSP3 | ROX | BHQ2 | 25 | 48 | 63 | 8829.1 |
| 9 | KLF2 | ATTO700 | BHQ1 | 22 | 59.1 | 64 | 8000.8 |
| 10 | ASUN | ATTO700 | BHQ1 | 26 | 46.2 | 63.2 | 9379.8 |
| 11 | NEMF | ROX | BHQ2 | 24 | 50 | 62.7 | 8623 |
| 12 | DHX29 | HEX | BHQ1 | 26 | 50 | 64.8 | 9079.6 |
| 13 | PTPRC | FAM | BHQ1 | 25 | 48 | 63 | 8767 |
| 14 | ZNF296 | FAM | BHQ1 | 24 | 54.2 | 64.4 | 8416.8 |
| 15 | ARG1 | ROX | BHQ2 | 24 | 54.2 | 64.4 | 8639 |
| 16 | ACTB | HEX | BHQ1 | 24 | 50 | 62.7 | 8789.6 |

***Supplementary Figure 2: Summarising the sample analysis protocol***


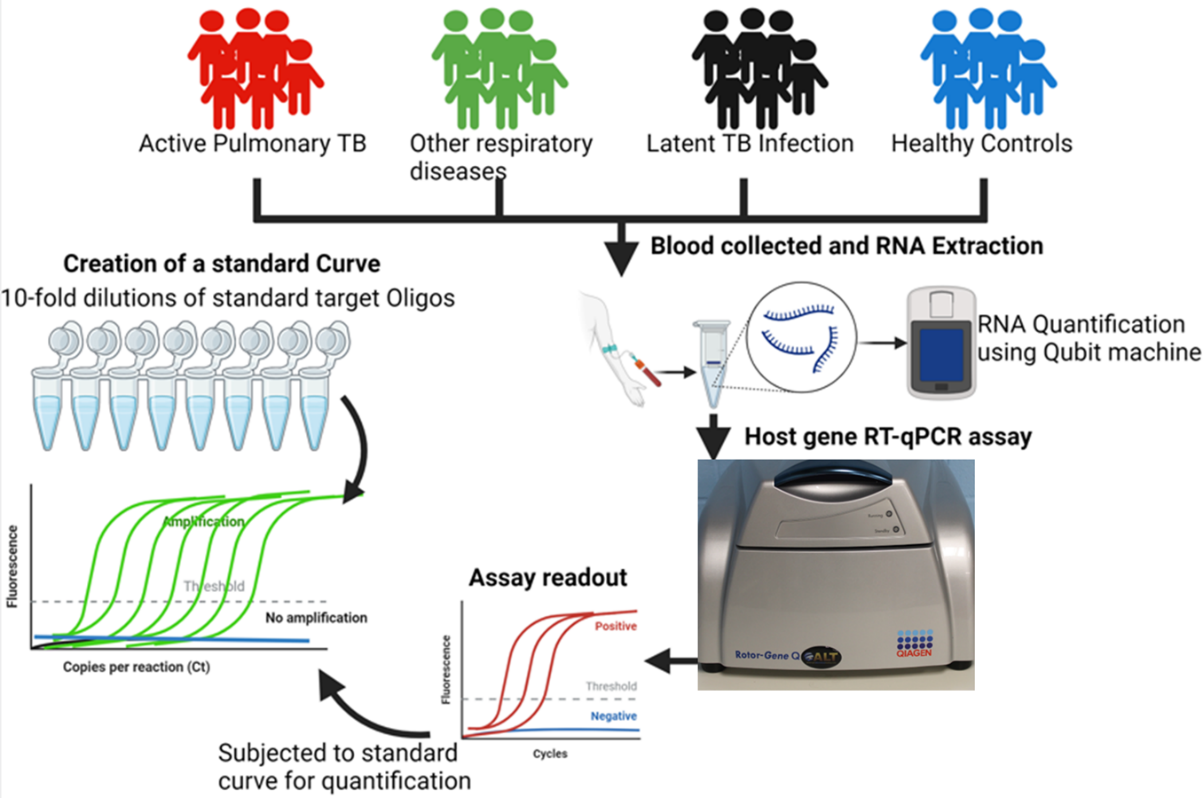


An outline of the whole protocol from sample collection to RT-qPCR result analysis. Blood was collected from all enrolled participants including active pulmonary TB (ATB), other respiratory diseases (ORDs), latent TB infection (LTBI) and healthy controls (HC) participants. The extracted RNA sample was initially quantified using Qubit, diluted (1:10) and amplification was performed on a Rotor-Gene Q machine. Two replicates were performed for each sample. All results were converted to copies/µl using the same specific target standard curve.

**References:**

1. Krapp, C. *et al.* Guanylate Binding Protein (GBP) 5 Is an Interferon-Inducible Inhibitor of HIV-1 Infectivity Article Guanylate Binding Protein (GBP) 5 Is an Interferon-Inducible Inhibitor of HIV-1 Infectivity. *Cell Host Microbe* **19**, 504–514 (2016).

2. Pilla-Moffett, D., Barber, M. F., Taylor, G. A. & Coers, J. Interferon-Inducible GTPases in Host Resistance, Inflammation and Disease. *J. Mol. Biol.* **428**, 3495–3513 (2016).

3. Shenoy, A. R. *et al.* GBP5 Promotes NLRP3 Inflammasome Assembly and Immunity in Mammals. *Science (80-. ).* **336**, 481 LP – 485 (2012).

4. Liu, P. F. *et al.* Guanylate-binding protein 6 is a novel biomarker for tumorigenesis and prognosis in tongue squamous cell carcinoma. *Clin. Oral Investig.* **24**, 2673–2682 (2020).

5. Keyse, S. M. Protein phosphatases and the regulation of mitogen-activated protein kinase signalling. *Curr. Opin. Cell Biol.* **12**, 186–192 (2000).

6. Guler, R., Roy, S., Suzuki, H. & Brombacher, F. Targeting Batf2 for infectious diseases and cancer. *Oncotarget* **6**, 26575–26582 (2015).

7. Hulett, M. D. & Hogarth, P. M. The second and third extracellular domains of FcγRI (CD64) confer the unique high affinity binding of IgG2a. *Mol. Immunol.* **35**, 989–996 (1998).

8. Tamoutounour, S. *et al.* CD64 distinguishes macrophages from dendritic cells in the gut and reveals the Th1-inducing role of mesenteric lymph node macrophages during colitis. *Eur. J. Immunol.* **42**, 3150–3166 (2012).

9. Repp, R. *et al.* Neutrophils Express the High Affinity Receptor for IgG (FcyRI, CD64) After In Vivo Application of Recombinant Human Granulocyte Colony-Stimulating Factor. *Blood* **78**, 885–889 (1991).

10. Perussia, B., Dayton, E. T., Lazarus, R., Fanning, V. & Trinchieri, G. Immune interferon induces the receptor for monomeric IgGl on human monocytic and myeloid cells. *J. Exp. Med.* **158**, 1092–1113 (1983).

11. Pearson, R., Fleetwood, J., Eaton, S., Crossley, M. & Bao, S. Krüppel-like transcription factors: A functional family. *Int. J. Biochem. Cell Biol.* **40**, 1996–2001 (2008).

12. Jha, P. & Das, H. KLF2 in regulation of NF-κB-mediated immune cell function and inflammation. *Int. J. Mol. Sci.* Nov 10;**18 (1):2383**, (2017).

13. Wu, G. *et al.* Molecular insights of Gas6/TAM in cancer development and therapy. *Cell Death Dis.* **8**, 1–10 (2017).

14. Morizono, K. *et al.* The Soluble serum protein gas6 bridges virion envelope phosphatidylserine to the TAM receptor tyrosine kinase Axl to mediate viral entry. *Cell Host Microbe* **9**, 286–298 (2011).

15. Meertens, L. *et al.* The TIM and TAM families of phosphatidylserine receptors mediate dengue virus entry. *Cell Host Microbe* **12**, 544–557 (2012).

16. Wu, S. *et al.* Septin4 promotes cardiomyocytes apoptosis by enhancing the VHL-mediated degradation of HIF-1α. *Cell Death Discov.* Jul 1; **7 (1): 172**, (2021).

17. Janeway, CA; Travers, P; Walport, M. The complement system and innate immunity - Immunobiology - NCBI Bookshelf. *Immunobiology: The Immune System in Health and Disease.* vol. 5th Editio 1–13 at https://www.ncbi.nlm.nih.gov/books/NBK27100/ (2001).

18. Jodoin, J. N. *et al.* Human Asunder promotes dynein recruitment and centrosomal tethering to the nucleus at mitotic entry. *Mol. Biol. Cell* **23**, 4713–4724 (2012).

19. Shao, S., Brown, A., Santhanam, B. & Hegde, R. S. Structure and assembly pathway of the ribosome quality control complex. *Mol. Cell* **57**, 433–444 (2015).

20. Filbeck, Sebastian, Cerullo, F., Pfeffer, S. & Claudio, A.P., J. Review mechanisms from bacteria to humans. *Mol. Cell* **82**, 1451–1466 (2022).

21. Parsyan, A. *et al.* The helicase protein DHX29 promotes translation initiation, cell proliferation, and tumorigenesis. *Proc. Natl. Acad. Sci. U. S. A.* **106**, 22217–22222 (2009).

22. Pisareva, V. P. & Pisarev, A. V. DHX29 reduces leaky scanning through an upstream AUG codon regardless of its nucleotide context. *Nucleic Acids Res.* **44**, 4252–4265 (2016).

23. Du, Y. & Grandis, J. R. Receptor-type protein tyrosine phosphatases in cancer. *Chin. J. Cancer* **34**, 61–69 (2015).

24. Kung, C. *et al.* Table 1 Peripheral Blood Lymphocyte Subpopulations. *Nat. Med. • Vol.* **6**, 343–345 (2000).

25. Guo, G., Li, B., Li, Q., Li, C. & Guo, D. PTPRC Overexpression Predicts Poor Prognosis and Correlates with Immune Cell Infiltration in Pediatric Acute Myeloid Leukemia. *Clin. Lab.* July 1;**68 (7)**, (2022).

26. Matsuura, T., Miyazaki, S., Miyazaki, T., Tashiro, F. & Miyazaki, J. ichi. Zfp296 negatively regulates H3K9 methylation in embryonic development as a component of heterochromatin. *Sci. Rep.* **7**, 1–12 (2017).

27. El Kasmi, K. C. *et al.* Toll-like receptor-induced arginase 1 in macrophages thwarts effective immunity against intracellular pathogens. *Nat. Immunol.* **9**, 1399–1406 (2008).

28. Pessanha, A. P., Martins, R. A. P., Mattos-Guaraldi, A. L., Vianna, A. & Moreira, L. O. Arginase-1 expression in granulomas of tuberculosis patients. *FEMS Immunol. Med. Microbiol.* **66**, 265–268 (2012).
